# Book Reviews

**Published:** 1903-11

**Authors:** 


### BOOK REVIEWS.

Surgical Diseases of the Abdomen. With special reference to diagnosis. By Richard Douglas, M. D. Formerly professor of Gynecology and Abdominal Surgery, Medical Department, Vanderbilt University, Nashville. Octavo, pp. 883. Illustrated by 20 full-page plates. Philadelphia: P. Blakiston's Son & Co. 1903. (Price, cloth, gilt top, $7.00.)

The importance of a thorough understanding of those diseases of the abdomen which may require surgical intervention, was never so insistent as at the present moment. Indeed, almost every abdominal affection may come within surgical purview at some stage in its course,—a fact that physicians, as distinguished from surgeons, have been somewhat slow to admit, but cannot further or longer dispute. The treatise before us will do much to clear the atmosphere of many doubts that have lead to misunderstandings and miscomprehensions in the diagnosis and management of diseases of the abdomen.

Inasmuch as diseases affecting the peritoneum lie at the threshold of the subject dealt with in this book, it is appropriate that Professor Douglas should begin by considering peritonitis. The opening chapter discusses the subject from every surgical viewpoint, and disposes of the old teaching relating to primary peritonitis, which Douglas says is of such rare occurrence as to justify its elimination in the consideration of the general symptomatology. Not less forceful is this author in treating of tuberculous peritonitis, which he defines as a specific inflammation of the peritoneum, excited by the bacillus tuberculosis.

Passing over many excellent chapters that deal with ulcer of the stomach, sarcoma and carcinoma of that organ, and like conditions of the duodenum, stenosis of the pylorus, foreign bodies in the stomach, and the like, we come to intestinal obstruction, where we pause for comment, first, because of the importance of the topic; and, second, because our author is so clear and sound in dealing with it. This is a condition where promptitude of diagnosis is imperative. A few hours delay may result fatally, hence the surgeon must be armed with definite knowledge, quick perception, and faultless technic. This chapter abounds in interest and deserves careful study. It teachings are of the best regarding this condition, which often taxes the skill and energy of the surgeon to the extreme limit.

The next topic upon which we shall comment is appendicitis. And here, again, we find the author not wanting in handling a subject of supreme importance. He surveys the whole field with judicial calmness and conservative judgment; in short, Douglas is so completely at home with his theme that scarcely a point, even of minor importance, is omitted, and he has presented many aspects of it which, if not entirely new, are developed in a new light. It is a terse, logical and scientific canvassing of one of the most important questions connected with abdominal surgery. It is essentially a surgical disease and must be studied from the vantage ground of the surgeon,—a syllogistic argument that pervades this chapter. There is so much of interest said in the space assigned to appendicitis that it becomes a difficult matter to break away from its consideration, but we must pass on.

Gallstone disease is another subject that has assumed great surgical importance within the last few years. Douglas has given an elaborate dissertation on this topic, and his tables of differential diagnosis have been prepared with much care. The whole question is discussed in a clear fashion and all the strong light of recent experience is thrown upon the screen. Every form of operation is detailed and the appropriate application of each is pointed out. This chapter is a succinct account of the disease, with all its complications, and will naturally attract the careful attention of surgeons, but it should not be neglected by physicians who do not practise surgery, but who themselves must be prepared to advise in emergency cases that so frequently arise.

Diseases of the kidney have become an attractive field for the surgeon of late years, and almost all conditions from renal calculus to chronic Bright's disease, have been successfully dealt with by the surgeon's knife. Douglas is particularly interesting in discoursing upon calculus, wandering kidney and hydronephrosis, but he is also graphic in dealing with renal tuberculosis and tumors of the kidney. He is abreast of the art in all he writes, regarding the various manifestations of kidney disease, and puts a vast deal of information into compact and even comparatively limited space.

Gunshot and stab wounds of the intestines and other abdominal viscera command appropriate attention and adequate treatment. Douglas, himself, has had a large experience in this kind of surgery, and reports his cases in tabular form. Out of 21 cases of gunshot wounds of the abdomen, involving various viscera, he reports 8 recoveries,—a splendid showing, considering the serious nature of the injuries.

Fibromyoma of the uterus, neoplasms of the ovaries and broad ligaments, and ectopic gestation constitute a triad of subjects that serve to bring to a fitting close this admirable treatise. Uterine fibromata are presented with much address, and the chapter allotted to their consideration is one of the strongest in the book. Neoplasms of the ovaries and broad ligaments receive due consideration, while ectopic pregnancy is masterfully presented.

In presenting this brief commentary, we have selected a few of the salients of this excellent contribution to the literature of surgery. It is a pretentious volume, and we regret that our notice does it such indifferent justice. There are many other subjects of great interest and importance, which are handled with admirable skill by the distinguished author, but the limitations of this review preclude comment upon them. The work is unique; it abounds in useful information, presented in a practical way. It deals with each topic with a directness that is attractive. Its definitions of the various diseases are terse and exact, and it will serve to distinctly classify surgical diseases of the abdomen. Finally, it is a work that can be studied with pleasure by the scholar, for it is a model in rhetorical finish, and is faultless in diction. The abdominal surgeon will be sure to obtain it because it is an exposition of the best thought in his special field of work, while it is equally adapted to the needs of the physician who does not practise surgery, because it gives him the information which he most needs and which is not to be found elsewhere in such available form. We could wish that the book had been more elaborately illustrated but, in view of its other excellent qualities, we may trust to future editions to meet this feature of medical bookmaking, which has become wellnigh an imperative necessity.

---

Gynecology. A Textbook for Students and a Guide for Practitioners. By William R. Pryor, M. D., Professor of Gynecology in the New York Polyclinic Medical School; Attending Gynecologist to New York Polyclinic Hospital; Consulting Gynecologist to St. Vincent's, New York City and St. Elizabeth's Hospitals. Octavo, pp. 396, with 163 illustrations in the text. New York and London: D Appleton & Company. 1903. (Price, $3.50.)

This textbook differs from others on gynecology in several particulars. In the first place, the author has placed a limit upon the subjects presented, including only those that are strictly gynecological and upon which a teacher of the topic would usually lecture or demonstrate. Rare diseases are not described or illustrated, nor are operations considered that now have been abandoned; bibliography has been omitted; anatomy and microscopy have not been appealed to in particular, and the treatise in this manner has been simplified and made to embrace only such material as will interest the undergraduate and general practitioner of medicine.

Two general divisions have been made of the book, the first, dealing with diseases which are described, and the second, with operations which, in the main, are illustrated. Pryor gives general direction relating to examination that, for the most part, are practical, but we think him in error in attaching so little importance to the kneechest posture. When needed it has not been found difficult or embarrassing in our experience. The chapter on inflammations is most satisfactory in description and treatment, puerperal sepsis being especially well presented. In treating the subjects of distortions and displacements, the author has done splendid work. His comments on the use of the pessary deserve to be carefully studied and remembered.

Diseases of the vulva receive rather less consideration than their importance requires, but not so of diseases of the vagina, urethra, and bladder; yet, perhaps, the author is at his best when dealing with tumors of the ovary and uterus. He describes the operations well that relate to these conditions, among which are a number of original points in technic. Pryor is very interesting, too, in handling the subject of cancer of the uterus, and gives a number of excellent illustrations of its pathology, many of which are taken from Cullen's elaborate treatise.

The surgery of the perineum and cervix are among the well-described and equally well-illustrated topics set forth; indeed, perhaps it is not out of place to say that the author is at his best in this part of the book. His technic is clever and his drawings illustrating this work are the best we have seen. Operations for the cure of retrodeviations of the uterus,—that much disputed field,—are given direct attention, and the author's views are clearly set forth. Abdominal section, hysterectomy, salpingo-oöphorectomy and the care or management of patients after abdominal sections are presented in succession. The author uses the word "celiotomy" in this latter relation,—an absurd term that is not needed and adds further to an already overburdened vocabulary.

The chapter on hysterectomy for pelvic suppuration deserves the careful study of every gynecologist. It contains much that is helpful toward a clearer understanding of the difficult problems connected with this complicated subject. Pryor gives prominence in text and picture to vaginal hysterectomy in dealing with pelvic suppuration, but he differentiates with skill the conditions wherein it is applicable. A few minor topics, including anomalies of the female genital tract, bring this interesting book to a close. It is a pleasure to read it from beginning to end, because of the sustained interest that is maintained all through its pages.

---

A System of Physiologic Therapeutics. A practical Exposition of the Methods, other than Drug-giving, useful for the Prevention of Disease and in the Treatment of the Sick. Edited by Solomon Solis Cohen, A. M., M. D., Senior Assistant Professor of Clinical Medicine in Jefferson Medical College. Volume VIII. Rest, Mental Therapeutics, Suggestion. By Francis X. Dercum, M. D., Professor of Nervous and Mental Diseases in the Jefferson Medical College of Philadelphia. Octavo, 332 pages. Philadelphia: P. Blakiston's Son & Co. 1903. (Price for the set, $27.50 net.)

The subjects considered in this volume,—rest, mental therapeutics, suggestion,—are nowhere set forth in such detail, or as scientifically treated, as has been done here by Professor Dercum. The importance of rest in the treatment of disease has been once and again pointed out by previous writers,—notably over twenty years ago by Mr. Hilton, in his classic monograph on rest and pain. But here the nervous system, which is the first to suffer damage by overwork and especially by worry, is dealt with as nowhere else in this particular relationship.

The present volume is principally devoted to the systematic consideration of that method of treatment sometimes flippantly termed "rest cure." This is the treatment given in the majority of sanatoria which, very justly, in most instances, makes them popular; but in this treatise physicians will learn how to apply rest in a clinically scientific fashion and how to make it fit the etiologic, pathologic and diagnostic factors pertaining to diseased or fretted conditions.

Two chapters, constituting the second part of the book, are set apart for the presentation of the therapeutics of mental diseases, which abound with valuable thoughts on the prevention and treatment of insanity and special forms of mental disease. Dr. Dercum is a master in this field, and his observations are entitled to the weight of authority.

In the third part of the volume, suggestion is considered in the most scientific manner. It is stripped of its mystery and its valuable features are laid bare for the use of medical men who can employ it rightfully. This section should be carefully studied by every practising physician, without regard to special lines of professional pursuit.

This series has contained many volumes of great interest and value, but we doubt if any one of them heretofore issued has excelled this particular number in importance or scientific worth. Dr. Dercum is a student of his art, clear of perception, and accurate in recording his facts. Moreover, he writes in clear English, and does not juggle with words. One cannot fail to understand his meaning, or to realise that he is presenting only those statements of fact which observation has proven to be demonstrated truths.

---

Disease of the Pancreas. Its Cause and Nature. By Eugene L. Opie, M. D., Associate in Pathology in the Johns Hopkins University; Fellow of the Rockefeller Institute of Medical Research. Octavo, pp. 359. Illustrated. Philadelphia and London: J. B. Lippincott Company. 1903. (Price, $3.00.)

Not until recently has pancreatic disease attracted that attention which its importance merits. The author of this book has contributed in a large measure to our present knowledge of the subject, in society papers and magazine articles published during the last three or four years. The treatise he now offers to the profession is the most pretentious American book dealing with disease of the pancreas.

After a somewhat lengthy preface, the anatomy of the pancreas is taken up and the variations from the ordinary are pointed out. An excellent reproduction of the original drawing of Wirsung is given. Anomalies of the pancreas are described and then an interesting chapter on its histology is presented. Now, we come to a consideration of acute inflammation of the pancreas,—a chapter that is most complete in its material and method, pointing out most clearly the several varieties of the disease in question. The association of acute pancreatitis and cholelithiasis is graphically described in the history of a case studied by the author at an autopsy, where over 100 gallstones were found, varying in diameter from a half to one centimeter. The etiological relation between these two conditions,—acute pancreatitis and gallstone disease,—is fully considered, and so, too, are hemorrhagic and gangrenous pancreatitis.

Fat necrosis furnishes the topic for a most interesting and instructive chapter, in which the production of this condition by experimental methods is described, and the whole subject receives elaborate treatment. Next, chronic interstitial pancreatitis in its several varieties, is taken up and dealt with in much detail at considerable length. Hyaline degeneration of the pancreas furnishes material for an instructive chapter, while the pathology of diabetes mellitus constitutes a chapter that should be read by every practising physician. The symptoms and treatment of pancreatic disease is the subject of the last chapter in the book, and is a fitting finale to one of the choicest contributions to medical literature that has appeared in recent years.

It is a work that reflects credit upon American medicine and will be prized by every one fortunate enough to possess it.

---

INTERNATIONAL CLINICS. A Quarterly of Illustrated Clinical Lectures and especially prepared original Articles on Treatment, Medicine, Surgery, Neurology, Pediatrics, Obstetrics, Gynecology, Orthopedics, Pathology, Dermatology, Ophthalmology, Otology, Rhinology, Laryngology, Hygiene, and other topics of interest to Students and Practitioners, by leading members of the medical profession throughout the world. Edited by A. O. J. KELLY, A. M., M. D., Philadelphia, with the collaboration of Wm. Osler, M. D., Baltimore; John H. Müsser, M. D., Philadelphia; Jas. Stewart, M. D., Montreal; John B. Murphy, M. D., Chicago; Thomas M. Rotch, M. D., Boston; John G. Clark, M. D., Philadelphia: James J. Walsh, M. D., New York; J. W. Ballantyne, M. D., Edinburgh; John Harold, M. D., London; Edmund Landolt, M. D., Paris, and Richard Kretz, M. D., Vienna. With regular correspondents in Montreal, London, Paris, Berlin, Vienna, Leipsic, Brussels and Carlsbad. Volume II. Thirteenth series. 1903. Philadelphia: J. B. Lippincott Company. 1903. (Cloth, $2.00.)

The field covered by the "clinics" is unique and it is being well occupied. The subjects considered in this volume are, the summer diarrhea of children, six articles; disease of the pancreas, two articles; treatment, four articles; medicine, four articles; surgery, three articles; pediatrics, one article; obstetrics and gynecology, three articles; and ophthalmology, one article. Opie's contribution on pancreatic disease will attract attention. His book on the subject is reviewed elsewhere in this edition of the JOURNAL. Deaver and Müller make an excellent contribution to the surgery of the pancreas,—an article that should be read by all surgeons.

The book, as usual, is replete with papers and lectures of interest by distinguished teachers at home and abroad.

---

SURGICAL ASEPSIS. Especially adapted to operations in the home of the patient. By HENRY B. PALMER, M. D., Consulting Surgeon to the Central Maine General Hospital. Ninety illustrations. Pages 237; large 12mo. Philadelphia: F. A. Davis Company. 1903. (Price, $1.25 net.)

The tendency in recent years has been to send patients to a hospital, when operative work is demanded. This is an excellent plan when delay is permissible, or when transportation does not place the patient in greater jeopardy. Now and then, however, an exigency arises that demands operation on the spot at the home of the patient, or wherever the accident happens that causes surgical inquiry. Occasionally, too, surgical disease suddenly takes on grave conditions, preventing removal of the patient to hospital. It is to meet such conditions that this manual has been prepared.

The book contains ample directions for the preparation of aseptic material, as well as for creating a surgical environment, during home operating and aftertreatment. It will be found a useful guide for the family physician, who may have immediate charge of the home treatment of surgical cases after operation. And also, it will serve to remind the surgeon himself of certain conditions that may not be neglected. It would be well, too, for nurses to familiarise themselves with the subject-matter of this excellent manual.

---

Transactions of the Southern Surgical and Gynecological Association. Vol. XV. Annual meeting, held at Cincinnati, O., November 11, 12 and 13, 1902. W. D. Haggard, M.D., Secretary. Philadelphia: Wm. J. Dornan, Printer. 1903.

This volume is fully up to the standard established by this association, as regards quantity and quality of the material which it contains. It records the proceedings of the fifteenth annual meeting, which was presided over by the late William E. B. Davis, who was the founder and organiser of the association. The fifteen volumes of transactions, which have now been issued, will always remain a monument to the memory of this distinguished and ever-to-be lamented physician, who was an ornament to his profession and a useful citizen far beyond the ordinary or average man.

This volume teems with excellent papers and discussions. It is a fitting complement to its predecessors, and an inspiration for future work. The next meeting of the association will be held at Atlanta, Ga., December 15-17, 1903, under the presidency of Dr. J. Wesley Bovée, of Washington, D. C.

